# The Role of Ultra‐Low‐Dose Naloxone as an Additive in Anesthesia and Nerve Block: A Systematic Review

**DOI:** 10.1002/hsr2.73287

**Published:** 2026-09-25

**Authors:** Mohammadjavad Mehrabanian, Behrang Nooralishahi, Mehdi Dehghani Firoozabadi, Mohammad Sahebjam, Yekta Ghane, Homayoun Pishraft‐Sabet, Nazila Heidari, Akbar Shafiee

**Affiliations:** ^1^ Tehran Heart Center, Cardiovascular Diseases Research Institute Tehran University of Medical Sciences Tehran Iran; ^2^ School of Medicine, Tehran University of Medical Sciences Tehran Iran; ^3^ School of Medicine, Iran University of Medical Sciences Tehran Iran

**Keywords:** anesthesia, naloxone, nerve block, systematic review

## Abstract

**Background and Aims:**

In anesthesia and analgesia, opioids alone or in combination with local anesthetics (bupivacaine, lidocaine, and ropivacaine) have been investigated to prolong peripheral nerve blocks. Ultra‐low‐dose naloxone administration in local anesthetic solutions could lengthen sensory and motor blocks through enhanced opioid effects or direct antagonistic effects on the receptors. We aim to assess the role of ultra‐low‐dose naloxone as an additive in anesthesia duration, motor, and sensory block duration, visual analog scale (VAS) scores, opioid consumption, and the incidence of postoperative complications.

**Methods:**

A systematic search was conducted in PubMed/Medline, Ovid Embase, and Web of Science up to November 23rd, 2023, and 840 records were retrieved and screened. Accordingly, seven randomized controlled trials met the eligibility criteria and were included for data extraction. The National Institute of Health's (NIH) Quality Assessment Tool for Clinical Trials was used for critical appraisal of the studies.

**Results:**

The effects of ultra‐low‐dose naloxone on intravenous regional anesthesia and various nerve blocks (including axillary and supraclavicular brachial plexus, peribulbar, femoral, and intercostal nerve block) were assessed. The administration of naloxone at doses of 100 and 50 ng as an additive to bupivacaine, lidocaine, and ropivacaine was associated with longer postoperative analgesia, prolonged motor and/or sensory block, lower postoperative opioid or analgesic consumption, and lower VAS pain scores in several included studies. Besides, in most studies, postoperative complications did not significantly differ between the groups.

**Conclusion:**

The available evidence suggests that ultra‐low‐dose naloxone may be associated with prolonged postoperative analgesia and, in some studies, longer motor and/or sensory block duration and reduced postoperative opioid consumption. These findings should be interpreted as observations reported by individual studies rather than definitive treatment effects. Although no major safety concerns were identified, the available evidence is insufficient to establish the overall safety or efficacy of ultra‐low‐dose naloxone.

## HIGHLIGHTS

- Evidence indicates that the co‐administration of local anesthetics and opioids might enhance the duration of nerve blockade.
- Naloxone, in combination with bupivacaine, lidocaine, and ropivacaine, might be a potential additive for improving postoperative analgesia and reducing opioid use.
- Several included studies reported prolonged postoperative analgesia and longer motor and/or sensory block duration with ultra‐low‐dose naloxone.
- Most included studies reported no significant differences in postoperative complications between the naloxone and comparator groups.
- Limited sample sizes and substantial clinical heterogeneity precluded quantitative meta‐analysis and warrant further randomized trials.

**Abbreviations**
CI: confidence intervals
IV: intravenous
IVRA: intravenous regional anesthesia
PONV: postoperative nausea and vomiting
SAMPL: statistical analyses and methods in the published literature
TKA: total knee arthroplasty
VAS: visual analog scale

## 1 Introduction

Various medications, such as Propofol, Opioids, Benzodiazepines, Ketamine, and Dexmedetomidine, have been used to induce anesthesia, both alone and in combination. Combinations of drugs, rather than individual medications, may reduce the amount of drug consumption and the possibility of side effects [1]. In procedures requiring general anesthesia or monitored anesthesia care, intravenous opiates provide additional analgesia and sedation. Opium anesthesia is approved for use by the Food and Drug Administration (FDA) at nearly all stages of surgery, including preinduction use for chronic pain control, induction and maintenance of anesthesia, and reduction of postoperative pain and restlessness [2].

In anesthesia and analgesia, different additives have been tested for their ability to extend the duration and intensity of regional blocks [3, 4, 5, 6, 7]. Opioids have been used both independently and in combination with local anesthetics to prolong peripheral nerve blocks [3, 4, 6, 7]. Given that opioids have dose‐dependent side effects and analgesic effects, a multimodal approach to anesthesia and analgesia could mitigate these consequences [8].

In recent years, evidence suggests that low doses of naloxone might selectively block opioid excitatory effects [9]. The inclusion of naloxone in local anesthetic solutions is likely to extend nerve sensory and motor blocks through enhanced opioid effects or direct antagonistic effects on these excitatory receptors [6, 10, 11, 12]. Previous investigations have shown that adding low doses of naloxone to bupivacaine, lidocaine, and ropivacaine extended the duration of sensory and motor block time [6, 13, 14].

In the context of administering an ultra‐low dose of naloxone to a nerve sheath, concerns about neurotoxicity have been raised. However, animal experiments have demonstrated the safety of intrathecal naloxone administration [15, 16]. In clinical studies, naloxone was administered without incident in conjunction with epidural opioids to reduce the adverse effects of opioids, including nausea, vomiting, and pruritus [17, 18, 19, 20, 21, 22]. In this systematic review, we evaluated the effect of ultra‐low‐dose naloxone as an additive on the duration of anesthesia, motor, and sensory block duration, visual analog scale (VAS) scores, opioid consumption, and the incidence of postoperative complications.

## 2 Material and Methods

The current systematic review was registered in PROSPERO (Registration Number: CRD42023478288) and performed based on the preferred reporting item for systematic reviews and meta‐analysis (PRISMA) 2020 recommendations [23]. The checklists are available in supplementary documents (Tables S1 and S2).

### 2.1 Search Strategy

A comprehensive systematic search was conducted across three databases, PubMed/Medline, Ovid Embase, and Web of Science, from their inception until November 23rd, 2023. A full list of search terms, including keywords and MeSH terms, can be found in the supplementary documents (Table S3).

### 2.2 Eligibility Criteria and Study Selection

Randomized controlled trials with full text available in English were eligible for inclusion in this systematic review. The source populations eligible for this review were individuals of any age and gender who underwent anesthesia or nerve block and received ultra‐low‐dose naloxone (at nanogram doses) or a placebo as an additive. No restriction was applied on the type of surgery, patient population, and age group. Studies that used higher doses of naloxone, retrospective and prospective cohort studies, case‐control studies, case series, case reports, review articles, book chapters, guidelines, conference abstracts, and experimental studies (including in vitro/ex vivo or animal studies) were excluded from this review.

### 2.3 Data Extraction

The data extraction process for the eligible studies was independently conducted by three reviewers (N.H., Y.G., and H.P.). The process was as follows: (I) Extraction of study characteristics, including author, year, design, sample size, type of surgery, method of anesthesia or nerve block, duration, and dose of adjuvant naloxone. (II) Extraction of results, including outcome measurements, complications, and adverse events. A fourth reviewer or the corresponding author reviewed any discrepancies or disagreements during the data extraction process.

The data were reported in accordance with the *statistical analyses and methods in the published literature (SAMPL)* guidelines for transparent presentation of quantitative results, including measures of central tendency, variability, and significance testing [24].

For each trial, between‐group comparisons (ultra‐low‐dose naloxone vs. comparator) were abstracted as reported. Unless explicitly stated otherwise in the source article, hypothesis tests were treated as two‐sided with *α* = 0.05.

### 2.4 Risk of Bias Assessment

Three investigators (Y.G., N.H., and H.P.) independently evaluated the methodological quality of the included articles and assessed the risk of bias. For these assessments, we used the National Institute of Health (NIH) Quality Assessment Tool for Clinical Trials (refer to Table S4) [25]. The bias assessment of the included studies revealed a range of quality scores from 10 (indicating Fair quality) to 13 (indicating Good quality), out of a maximum possible score of 14.

## 3 Results

### 3.1 Search Results

**Figure 1:**
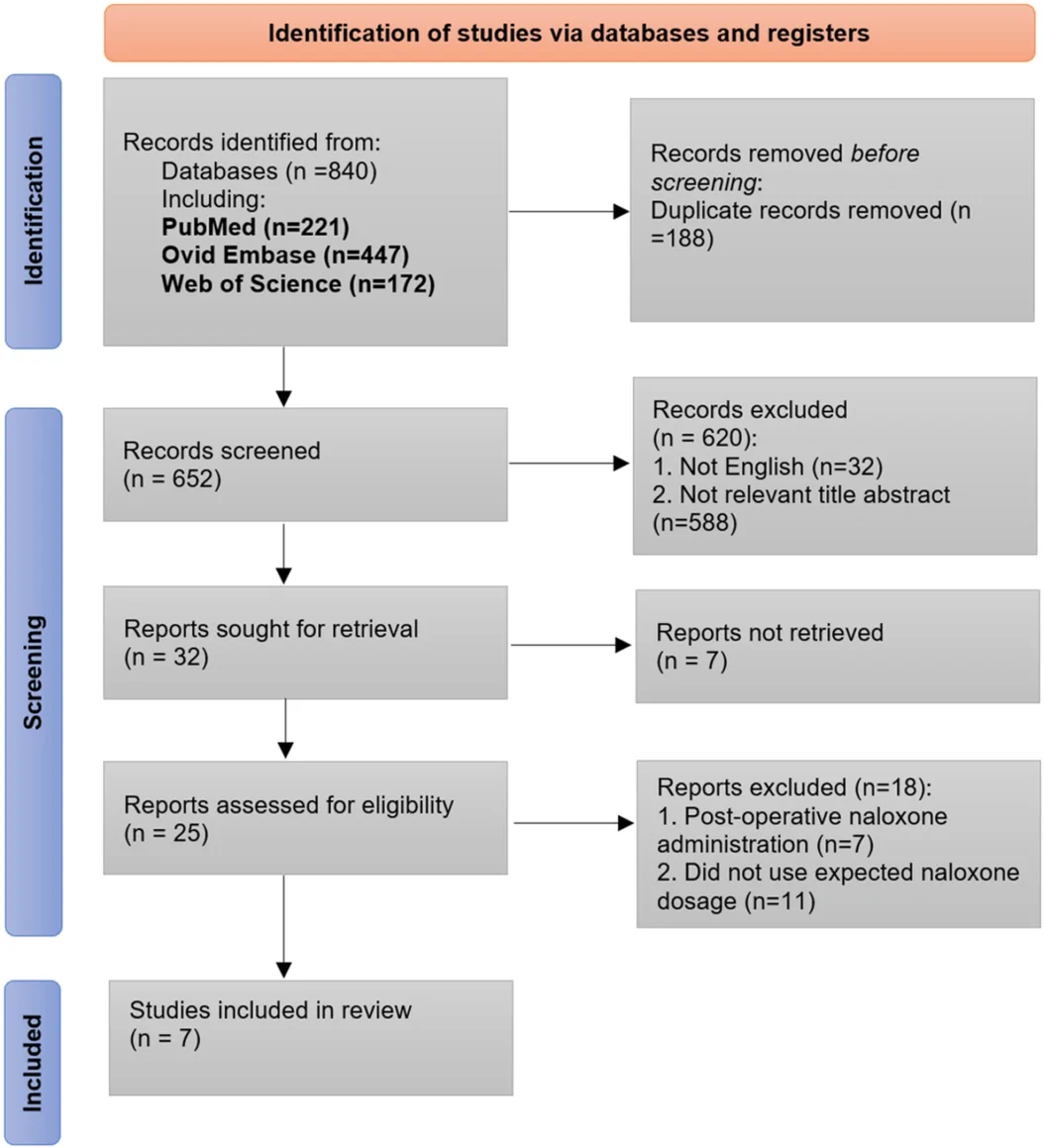
PRISMA 2020 flow diagram for new systematic reviews included searches of databases and registers only.

A total of 840 records were identified. Of these, 188 duplicates were detected and subsequently removed. The titles and abstracts of the remaining 652 articles were reviewed in the first and second steps of the screening process. Any disagreements that arose during this process were resolved through consensus with the corresponding author. In the final phase of the screening, the full texts of 25 studies were reviewed based on predefined eligibility criteria. Ultimately, seven articles were selected for data extraction. The PRISMA flowchart detailing the inclusion process is displayed in Figure 1.

### 3.2 Characteristics of Eligible Studies

Seven randomized controlled trials have evaluated the role of ultra‐low‐dose naloxone as an additive in nerve block and anesthesia in 564 adult individuals scheduled for surgery. All articles included in our systematic review focused on the adult population. Although no age restrictions were imposed during the search, all included studies were conducted exclusively in adult populations, and no pediatric patients were enrolled. In all studies, adjuvant naloxone was administered at a dose of 100 ng [6, 13, 14, 26, 27, 28], except for one study that administered doses of both 50 and 100 ng [29]. In our population, surgery on the forearm, arm, and hand was performed in 65.6% of the patients (*n* = 370). Other types of surgical procedures included total knee arthroplasty (TKA) (13.12%, *n* = 74), unilateral thoracic surgery (10.63%, *n* = 60), and open globe cataract surgery (10.63%, *n* = 60). The characteristics of eligible studies are illustrated in Table 1. Of the seven studies, only three involved blocks of large peripheral nerves, one (peribulbar) was a technique with multiple possible effects, one was with infiltration (intrapleural) and 2 were in intravenous regional anesthesia (IVRA).

**Table 1:** Characteristics of included studies utilizing ultra‐low‐dose naloxone in anesthesia or nerve block.

Study ID (author, date)	Study design	Sample size	Type of surgery	Method of anesthesia or nerve block	Dose of adjuvant naloxone	Duration of anesthesia or nerve block	Outcome measurement	Complications and adverse events
Lee, 2021 [13]	Double‐blind RCT	74	Primary unilateral TKA for OA or RA	Femoral nerve block G1 (*n* = 37): 20 mL of 0.375% ropivacaine with 1 mL of isotonic saline chloride G2 (*n* = 37): 20 mL of 0.375% ropivacaine with 100 ng of naloxone	100 ng	G1: 185.3 ± 13.7 min G2: 188.3 ± 12.3 min	Significantly longer elapsed time before the first request for analgesia in G2 (735.5 ± 187.2 min) than that in G1 (602.6 ± 210.4 min) A significantly lower total dose of supplementary opioids consumption was in G2 (312.4 ± 141.7 μg) than that in G1 (456.5 ± 279.5 μg) No difference in the onset time of sensory blockade between the two groups (G1: 22.8 ± 10.8, G2: 19.2 ± 6.3) Lower VAS scores for G1 than for G2 at rest and during activity of the knee (rest: 12 h and 18 h, activity: 12 h), no significant difference in the VAS scores between groups at 0, 6, and 24 h at rest and at 18 and 24 h during activity in the postoperative period	No local anesthetic toxicity or, neurological complications, or falls in either group No significant difference in incidences of complications in the two groups Hypotension: G1 (*n* = 3), G2 (*n* = 2) Nausea and vomiting: G1 (*n* = 6), G2 (*n* = 5) pruritus: G1 (*n* = 2), G2 (*n* = 2) Urinary retention: G1 (*n* = 7), G2 (*n* = 8)
Eissa, 2018 [29]	Double‐blind RCT	150	Elective forearm and arm surgery	Intravenous regional anesthesia G1: (Regional 100) (*n* = 30): lidocaine and naloxone (3 mg/kg of 2% lidocaine and naloxone 100 ng (1 mL) diluted with normal saline to 30 mL) G2: (Regional 50) (*n* = 30): lidocaine and naloxone (3 mg/kg of 2% lidocaine and naloxone 50 ng (1 mL) diluted with normal saline to 30 mL). G3: (Intravenous 0.25 μg/kg/h) (*n* = 30): lidocaine and intravenous naloxone infusion (3 mg/kg of 2% lidocaine diluted with normal saline to 30 mL will be given to the operating hand) and in the other hand naloxone infusion at a rate of (0.25 μg/kg/h). G4: (Intravenous 0.15 μg/kg/h) (*n* = 30): lidocaine and intravenous naloxone infusion (3 mg/kg of 2% lidocaine diluted with normal saline to 30 mL will be given to the operating hand) and in the other hand naloxone infusion at a rate of (0.15 μg/kg/h). Group V: (Control group) (*n* = 30): lidocaine (3 mg/kg of 2% lidocaine diluted with normal saline to 30 mL will be given to the operating hand) and in the other hand intravenous normal saline infusion	G1: 100 ng G2: 50 ng	Significantly shorter duration of onset of motor and sensory blocks in G1 and G2 compared to other groups (naloxone infusions and lidocaine alone)	Significantly longer duration of offset of motor and sensory blocks in G1 and G2 compared to other groups Significantly delayed onset of tourniquet pain was significantly delayed in G1 and G2 compared to other groups Significant reduction in VAS pain score at the end of surgery in all naloxone‐received patients than in patients who received lidocaine alone Less VAS pain score and amount of fentanyl consumption in G1 and G2 compared to other groups (naloxone infusions and lidocaine alone) No significant difference between different groups in follow‐up in terms of heart rate, mean arterial pressure, and SpO_2_	Safe with no recorded side effects
Amer, 2019 [26]	Double‐blind RCT	60	Unilateral thoracic surgery (diaphragmatic hernia repair, lymph node excision, decortication, and pneumonectomy)	Intercostal nerve block G1: 30 mL of 0.5% bupivacaine injected from an intrapleural catheter in 5 min while clamping the chest tube for 15 min (*n* = 30) G2: 30 mL of 0.5% bupivacaine with 100 ng of naloxone injected from intrapleural catheter over 5 min while clamping the chest tube for 15 min (*n* = 30)	100 ng	NA	Significant decrease in VAS score in G2 at 6 h and 8 h after the operation, then a significant increase after 12 h after injection Longer time for the first request of rescue analgesia in G2 (11 ± 0.8 h) compared to G1 (6.7 ± 0.9 h) Lower total amount of morphine consumed in G2 (13 ± 0.8 mg) than G1 (26.4 ± 1.1 mg)	No significant adverse event, no significant difference between groups G1: bradypnea (*n* = 1) and two vomiting (*n* = 2) G2: bradypnea (*n* = 1) and two vomiting (*n* = 1)
El‐Sayed, 2016 [27]	Double‐blind RCT	40	Elective hand and forearm surgery	Intravenous regional anesthesia G1 (*n* = 20): 3 mg/kg of 2% lidocaine diluted with normal saline to 30 mL G2 (*n* = 20): 3 mg/kg of 2% lidocaine and naloxone 100 ng (1 mL) diluted with normal saline to 30 mL	100 ng	Significantly longer recovery of sensory block in G2 (26.7 ± 2.8 min compared to G1 (16.3 ± 0.6 min) Significantly longer recovery of motor block in G2 (19.1 ± 1.0 min) compared to G1 (17.9 ± 1.2 min)	Significantly less intraoperative fentanyl requirement in G2 (15.8 ± 5.0 mcg) compared to G1 (40.0 ± 10.5 mcg) Significantly longer first fentanyl requirement in G2 (22.4 ± 3.1 min) compared to G1 (14.5 ± 6.1 min) Longer first postoperative analgesic requirement in G2 (78.5 ± 6.8 min) compared to G1 (40.5 ± 2.0 min) The significantly less total amount of diclofenac needed in 24 h in G2 (57 ± 50 mg) compared to G1 (120 ± 45 mg) Significant less VAS score in G2 than G1 at 30 min (1.2 ± 1.2, 2.5 ± 0.7) after tourniquet inflation and 15 (1.9 ± 1.7, 3.2 ± 1.9) and 240 (2.0 ± 0.9, 2.9 ± 1.2) minutes after release of tourniquet	NA
Marashi, 2015 [14]	Double‐blind RCT	68	Elective forearm and arm surgery	Supraclavicular brachial plexus block G1 (*n* = 17): 30 mL bupivacaine G2 (*n* = 17): 30 mL bupivacaine with 100 µg of fentanyl G3 (*n* = 17): 30 mL bupivacaine with 100 ng naloxone G4 (*n* = 17): 30 mL bupivacaine with 100 µg of fentanyl and 100 ng naloxone	100 ng	Duration of sensory and motor block: G1: 11.3 ± 1.7 h and 4.56 ± 1.0 h G2: 12.8 ± 3.3 h and 5.1 ± 2.0 h G3: 18.1 ± 2.2 h and 6.18 ± 1.0 h G4: 15.8 ± 2.9 h and 6.53 ± 1.1 h	The same sensory and motor onset times in all groups More morphine requirement in G1 (28.2 ± 10 mg) than other groups (G2: 20.3 ± 7, G3: 22.2 ± 4, G4: 19.7 ± 5) Same VAS score in all groups	Higher incidence of PONV in G2 (58.85%) than in G1 (17.6%), G3 (29.4%), or G4 (30.1%) Higher incidence of pruritus in G2 (41.2%) than G1 (17.6%), G3 (11.8%), and G4 (11.8%)
Ezz, 2015 [28]	Double‐blind RCT	60	Open globe cataract surgery	Peribulbar block Group 1 (*n* = 30): fentanyl group, administration of 50 μg fentanyl and lidocaine 2% with hyaluronidase 15 IU/mL G2 (*n* = 30): fentanyl‐naloxone group, 50 μg fentanyl and lidocaine 2% with hyaluronidase 15 IU/mL, plus naloxone Topical local anesthetic with benoxinate hydrochloride for both groups	100 ng	G1: 51.93 ± 3.81 min G2: 53.80 ± 3.89 min	Quality of block: no significant difference in the quality of block regarding the total injected volume (8.47 ± 1.33 mL in G1 vs. 9.00 ± 1.95 mL in G2), onset (8.40 ± 2.84 min in G1 vs. 8.13 ± 2.47 in G2), best akinesia score (15.83 ± 3.04 in G1 vs. 16.67 ± 2.60 in G2), and number of patients needed supplemental injection (5 patients in G1 vs. 4 patients in G2) Significant longer time to first rescue analgesic in G2 (7.73 ± 0.98 h) compared to G1 (4.30 ± 0.47 h) Significant lower VAS score at 60, 90 min, 2, and 3 h, then increased significantly at 6 h postoperatively in G2 compared to G1 No significant difference in the total akinesia score at 2, 4, 6, 8, and 10 min postinjection between the two groups	Chemosis: G1 (*n* = 1), G2 (*n* = 2) Pain on injection: G1 (*n* = 3), G2 (*n* = 2)
Movafegh, 2009 [6]	Double‐blind RCT	112	Elective forearm surgery: CRPF: G1 (*n* = 19), G2 (*n* = 16), G3 (*n* = 18), G4 (*n* = 17) ORIF: G1 (*n* = 4), G2 (*n* = 5), G3 (*n* = 5), G4 (*n* = 4) P&P: G1 (*n* = 3), G2 (*n* = 4), G3 (*n* = 3), G4 (*n* = 3)	Axillary brachial plexus block G1 (*n* = 28): control group, 34 mL lidocaine 1.5% with 3 mL of isotonic saline chloride G2 (*n* = 28): fentanyl group, 34 mL lidocaine 1.5% with 2 mL (100 μg) of fentanyl and 1 mL of isotonic saline chloride G3 (*n* = 28): naloxone group, 34 mL lidocaine 1.5% with 2 mL saline chloride and naloxone G4 (*n* = 28): naloxone + fentanyl group, 34 mL lidocaine 1.5% with 2 mL (100 μg) of fentanyl and naloxone	100 ng	cG1: 38.0 ± 11.9 min fG2: 37.7 ± 11.6 min nG3: 43.4 ± 13.0 min nfG4: 42.7 ± 11.9 min	Significant longer sensory onset time in G3 (15 ± 3) and G4 (17 ± 3) compared to G1 (10 ± 3) and G2 (10 ± 4) Significant longer motor onset time in G3 (21 ± 4) and G4 (17 ± 3) compared to G1 (15 ± 5) and G2 (14 ± 7) Significant longer duration of time to first postoperative pain in G3 (92 ± 10) and G4 (98 ± 12) compared to G1 (68 ± 7) and G2 (68 ± 11) Significant longer duration of motor blockade in G3 (115 ± 10) and G4 (122 ± 16) compared to G1 (89 ± 11) and G2 (90 ± 12)	PONV: G1 (*n* = 1), G2 (*n* = 2), G3 (*n* = 1) Pruritis: G1 (*n* = 4), G2 (*n* = 4), G3 (*n* = 2), G4 (*n* = 2) No significant differences in the incidence of PONV and pruritus

Abbreviations: CRPF, closed reduction percutaneous fixation; G, group; h, hour; min, minute, OA, osteoarthritis; ORIF, open reduction internal fixation; P&P, pin and plaster; PONV, postoperative nausea and vomiting; RA, rheumatoid arthritis; RCT, randomized control trial; TKA, total knee arthroplasty; VAS, visual analog scale.

Due to the clinical heterogeneity in block types, anesthetic agents, and outcome reporting (peripheral vs. regional vs. infiltration), formal meta‐analysis was not feasible. Instead, results are presented as a narrative synthesis with quantitative effect estimates in accordance with the SAMPL guidelines. Efforts were made to extract effect sizes and 95% confidence intervals (CIs) from each included study to improve the clinical interpretability of results. However, most of the included randomized controlled trials reported only mean ± standard deviation and P‐values, without accompanying CIs or standardized effect sizes. If available, the reported CIs were recorded directly. For studies lacking these metrics, descriptive quantitative comparisons (mean differences, direction, and magnitude of effect) were summarized without attempting to impute or reconstruct missing CIs to avoid introducing bias. All statistical results were reported using two‐sided significance thresholds (*α* = 0.05).

#### 3.2.1 Nerve Block

Five articles involving 374 patients evaluated the impact of adding 100 ng of naloxone to ropivacaine, lidocaine, and bupivacaine in axillary and supraclavicular brachial plexus, peribulbar, femoral, and intercostal nerve blocks. Movafegh and colleagues assessed the effect of adding 100 ng naloxone to lidocaine or a mixture of lidocaine and fentanyl on the onset and duration of axillary brachial plexus block [6]. Sensory and motor onset times were longer in the naloxone (sensory onset 15 ± 3 min, motor onset 21 ± 4 min) and naloxone + fentanyl groups than in the control or fentanyl groups (sensory onset 10 ± 3 min in the control group, 10 ± 4 min in the fentanyl group, and 17 ± 3 min in the naloxone + fentanyl group; motor onset 15 ± 5 min in control, 14 ± 7 min in fentanyl, and 17.3 ± 3.4 min in naloxone + fentanyl; *p* < 0.001). The duration of time to first postoperative pain and motor blockade was significantly longer in the naloxone (92 ± 10 min and 115 ± 10 min, respectively) and naloxone + fentanyl groups (98 ± 12 min and 122 ± 16 min) compared with the control (68 ± 7 min and 89 ± 11 min) and fentanyl groups (68 ± 11 min and 90 ± 12 min) (*p* < 0.001, one‐way ANOVA, Tukey post hoc, two‐tailed). The time to first postoperative pain was therefore significantly longer in both naloxone‐containing groups compared with control or fentanyl alone (*p* < 0.001). No differences were observed in the incidence of postoperative nausea and vomiting (PONV) or pruritus.

Marashi et al. conducted a randomized, double‐blind clinical trial of 68 patients undergoing elective upper‐limb orthopedic surgery under supraclavicular brachial plexus block [14]. Participants were assigned to four groups (*n* = 17 each): G1—30 mL 0.5% bupivacaine (control); G2—bupivacaine + 100 µg fentanyl; G3—bupivacaine + 100 ng naloxone; and G4—bupivacaine + 100 µg fentanyl + 100 ng naloxone. Onset times for sensory and motor block did not differ significantly among groups (*p* = 0.67 and 0.54, respectively). The duration of sensory block was significantly prolonged in the naloxone groups (18.1 ± 2.2 h in G3 and 17.8 ± 2.4 h in G4) compared with the control (11.3 ± 3.0 h) and fentanyl groups (12.3 ± 2.4 h) (*p* < 0.001; one‐way ANOVA, Tukey post hoc, two‐tailed). Similarly, the duration of motor block was longer in the naloxone groups (6.18 ± 1.0 h in G3 and 6.03 ± 1.2 h in G4) than in G1 (4.56 ± 1.0 h) and G2 (4.83 ± 0.9 h) (*p* < 0.001). Postoperative morphine consumption within 24 h was also lower in the naloxone groups (9.2 ± 1.6 mg in G3 and 9.5 ± 1.8 mg in G4) than in G1 (14.3 ± 2.1 mg) and G2 (13.7 ± 2.0 mg) (*p* < 0.001). VAS pain scores did not differ significantly during the first eight postoperative hours (*p* = 0.58). The incidence of postoperative nausea and vomiting (PONV) and pruritus was higher in the fentanyl‐containing groups (G2 and G4) than in the naloxone‐only and control groups, but this difference was not significant.

Ezz and Elkala performed a randomized, double‐blind trial involving 60 adult patients undergoing open‐globe cataract surgery under peribulbar anesthesia [28]. Patients were allocated to Group 1 (50 µg fentanyl + 2% lidocaine + hyaluronidase 15 IU/mL) or Group 2, which received the same mixture supplemented with 100 ng naloxone. The time to first rescue analgesic was significantly longer in the naloxone group (7.73 ± 0.98 h) than in controls (4.30 ± 0.47 h; *p* < 0.001, independent two‐tailed *t*‐test). There were no significant differences between groups in total injected volume (8.47 ± 1.33 mL vs. 9.00 ± 1.95 mL; *p* = 0.221), onset time (8.40 ± 2.84 min vs. 8.13 ± 2.47 min; *p* = 0.699), or best akinesia score (15.83 ± 3.04 vs. 16.67 ± 2.60; *p* = 0.259). VAS pain scores were significantly lower in the naloxone group at 60, 90 min, 2 h, and 3 h postoperatively (*p* ≤ 0.003). Intraocular pressure rose transiently at 2 min postinjection but returned to baseline by 10 min in both groups. Minor complications such as chemosis and injection‐site pain occurred at similar frequencies (6.7% vs. 3.3% and 6.7% vs. 10%, respectively; *p* > 0.05). No cases of diplopia, globe perforation, or systemic adverse effects were reported.

Amer et al. used a small dose of naloxone as an adjuvant to bupivacaine in intrapleural infiltration following thoracotomy surgery [26]. Patients were divided into two equal groups: Group 1, 20 mL of 0.25% bupivacaine (control); and Group 2, 20 mL of 0.25% bupivacaine with 100 ng naloxone administered intrapleurally after skin closure. VAS scores were significantly lower in the naloxone group at 6 and 8 h postoperatively (*p* < 0.05), but differences were not sustained at 12 h. The time to first request for rescue analgesia was longer in the naloxone group (6.8 ± 1.2 h) than in controls (5.3 ± 1.1 h; *p* = 0.002, two‐tailed *t*‐test). In addition, 24‐h morphine consumption was lower with naloxone (5.8 ± 1.4 mg) than control (7.6 ± 1.9 mg; *p* < 0.01), a reduction of approximately 25%. No differences were noted in vital signs or postoperative complications (*p* > 0.05).

In a clinical trial by Lee et al., ropivacaine alone or combined with 100 ng naloxone was used for femoral nerve block in 74 patients scheduled for unilateral TKA [13]. Participants were randomized into two equal groups: G1 (*n* = 37) received 20 mL of 0.375% ropivacaine with 1 mL of isotonic saline, and G2 (*n* = 37) received 20 mL of 0.375% ropivacaine with 100 ng of naloxone diluted in 1 mL saline. The time to first rescue analgesia was significantly longer in G2 (735.5 ± 187.2 min) than in G1 (602.6 ± 210.4 min; *p* = 0.003). The total opioid consumption within 24 h was significantly lower in G2 (312.4 ± 141.7 µg) than in G1 (456.5 ± 279.5 µg; *p* = 0.007). Postoperative VAS pain scores at rest were lower in G2 at 12 h (*p* = 0.001) and 18 h (*p* = 0.043), and during knee activity at 12 h (*p* = 0.001). No significant differences were found in the onset or duration of sensory block (188.3 ± 12.3 min in G1 vs. 185.3 ± 13.7 min in G2; *p* > 0.05) or motor block duration (242.2 ± 24.8 min vs. 239.3 ± 22.5 min; *p* > 0.05). Hemodynamic variables, patient satisfaction, and adverse events (hypotension, PONV, pruritus, urinary retention) did not differ significantly between groups.

#### 3.2.2 IVRA

Two studies involving a total of 190 participants undergoing elective forearm and arm surgery investigated the effect of adding ultra‐low‐dose naloxone as an adjuvant to lidocaine for IVRA.

In the first study, El‐Sayed et al. performed a prospective, randomized, double‐blind trial in 40 patients undergoing short‐hand or forearm procedures under IVRA [27]. Participants were equally allocated to two groups: G1 (*n* = 20) received 3 mg/kg of 2% lidocaine diluted with normal saline to 30 mL, whereas G2 (*n* = 20) received 3 mg/kg of 2% lidocaine with 100 ng naloxone (1 mL) diluted with normal saline to 30 mL.

Compared with the control group, the addition of naloxone significantly improved several intra and postoperative parameters. The recovery of sensory block was longer in G2 (26.7 ± 2.8 min) than in G1 (16.3 ± 0.6 min; *p* < 0.001), and the recovery of motor block was also prolonged (19.1 ± 1.0 min vs. 17.9 ± 1.2 min; *p* = 0.002). Furthermore, patients in the naloxone group required less intra‐operative fentanyl (15.8 ± 5.0 vs. 40.0 ± 10.5 µg; *p* < 0.001) and had a longer time to first fentanyl request (22.4 ± 3.1 vs. 14.5 ± 6.1 min; *p* < 0.001). Postoperatively, the time to first analgesic requirement was extended (78.5 ± 6.8 vs. 40.5 ± 2.0 min; *p* < 0.001), and the 24‐h diclofenac consumption was significantly reduced (57 ± 50 vs. 120 ± 45 mg; *p* < 0.001). In addition, VAS pain scores were consistently lower in the naloxone group at all postoperative time points (*p* < 0.05). Hemodynamic parameters and satisfaction scores did not differ significantly between groups, and no adverse events were reported.

Eissa et al. conducted a randomized, double‐blind, controlled study involving 150 patients undergoing short upper‐limb surgeries under IVRA [29]. Patients were randomly assigned to five equal groups (*n* = 30 each):

G1 (Regional 100): 3 mg/kg of 2% lidocaine and naloxone 100 ng (1 mL) diluted with normal saline to 30 mL; G2 (Regional 50): 3 mg/kg of 2% lidocaine and naloxone 50 ng (1 mL) diluted with normal saline to 30 mL; G3 (Intravenous 0.25 µg/kg/h): 3 mg/kg of 2% lidocaine diluted with normal saline to 30 mL administered to the operative limb, and an IV naloxone infusion at 0.25 µg/kg/h via the contralateral arm; G4 (Intravenous 0.15 µg/kg/h): 3 mg/kg of 2% lidocaine diluted with normal saline to 30 mL administered to the operative limb, and an IV naloxone infusion at 0.15 µg/kg/h via the contralateral arm; G5 (Control): 3 mg/kg of 2% lidocaine diluted with normal saline to 30 mL administered to the operative limb, and an IV infusion of normal saline in the other arm.

The results showed that the addition of ultra‐low‐dose naloxone as a local adjuvant significantly improved block characteristics compared with control. The onset time of sensory block was shortest in G1 (Regional 100) (4.9 ± 1.2 min) and G2 (Regional 50) (5.2 ± 1.1 min) compared with control (G5, 6.5 ± 1.3 min; *p* < 0.05). Similarly, the onset of motor block was faster in the local naloxone groups (6.1 ± 1.4 and 6.5 ± 1.5 min) than in the control (7.8 ± 1.3 min; *p* < 0.05). The recovery time of both sensory and motor block was significantly prolonged in G1 (36.5 ± 3.6 and 31.2 ± 3.1 min) and G2 (33.7 ± 3.8 and 29.8 ± 2.7 min) compared with control (24.1 ± 2.9 and 22.5 ± 3.1 min; *p* < 0.05). Moreover, the time to first postoperative analgesic request was significantly longer in G1 (138.2 ± 14.7 min) and G2 (125.6 ± 13.8 min) compared with G5 (93.7 ± 11.9 min; *p* < 0.05). VAS pain scores at 30, 60, and 120 min were significantly lower in the regional naloxone groups than in the control group (*p* < 0.05). In contrast, intravenous naloxone infusion (G3 and G4) did not produce significant differences from the control group in any of the measured parameters, including onset, recovery, VAS scores, or postoperative analgesic consumption (*p* > 0.05). No cardiovascular, neurological, or local complications were observed in any group, and hemodynamic stability was maintained throughout the study.

## 4 Discussion

This study systematically evaluated the efficacy of administering ultra‐low‐dose naloxone as an additive in nerve block and anesthesia in 564 individuals undergoing surgery. The focus was on the duration of anesthesia, motor and sensory block duration, VAS scores, opioid consumption, and the incidence of postoperative complications. Overall, our findings highlighted that adding naloxone to bupivacaine, lidocaine, and ropivacaine at doses of 100 and 50 ng might be associated with prolonged motor and sensory block duration. Additionally, naloxone might reduce opioid consumption and VAS scores when prescribed in small doses. Besides, most studies did not reveal a significant difference between the naloxone‐received groups and other groups in terms of postoperative complications.

Regional anesthesia is a subgroup of anesthesia that involves using anesthetic medication to block sensation in a specific area of the body, such as during a peripheral nerve block [30]. Moreover, regional anesthesia allows patients to breathe spontaneously during an operative or other invasive procedure. This approach reduces pulmonary complications resulting from general anesthesia and enhances postoperative recovery. The most common medications for regional anesthesia include procaine, tetracaine, lidocaine, mepivacaine, prilocaine, bupivacaine, and ropivacaine. Neuraxial anesthesia, peripheral nerve blocks, and IVRA are the three main types of regional anesthesia. These should be considered the preferred anesthetic methods for patients at high risk of respiratory distress due to general anesthesia, as well as for those who wish to avoid systemic medications [31, 32].

There has been a growing consensus on the co‐administration of local anesthetics and opioids to enhance the effectiveness and duration of nerve blockades in recent decades. Certain receptors play a role in pain sensation along peripheral sensory nerve fibers. When opioids are administered peripherally, they exhibit pain‐relieving properties; however, the efficacy of opioids on regional blockade is still debatable [6]. Some researchers have reported superior results and postoperative analgesia after brachial plexus block with opioids [33, 34, 35], while others have found no significant effect from using opioids in nerve block [7, 36]. It is important to emphasize that using opioids for anesthesia can increase the likelihood of respiratory failure and postsurgery adverse events such as hyperalgesia, nausea, vomiting, itching, ileus, constipation, urinary retention, tolerance by desensitization, vertigo, and drowsiness [37]. Therefore, it's crucial to choose an additive medication in regional anesthesia that can enhance nerve block duration and decrease intraoperative/postoperative opioid dosage to limit unwanted consequences.

Overall, opioids can exert both excitatory and inhibitory modulation of sensory neurons' action potentials [6]. Naloxone is a well‐known antidote to opioid agents typically prescribed to reverse opioid overdose consequences [38]. Naloxone has demonstrated paradoxical results: it acts as an antagonist to opioid analgesia when administered in high doses (in the microgram range) but generates an anti‐nociceptive response when given in extremely low doses (in the nanogram range) [39]. Naloxone and naltrexone exhibit specific antagonistic properties on the stimulatory functions of opioid receptors in the nociceptive dorsal root ganglion [40]. This mechanism reveals the pain‐relieving capabilities of morphine and other opioids affecting various types of receptors (*μ*, *κ*, *σ* receptors). This impact is developed in response to ultra‐low doses of naloxone and naltrexone, which extend the Ca2 ± dependent portion of the nerve cell action potential. Moreover, through high‐affinity interactions between naloxone and a penta‐peptide region of the C‐terminal filamin A, which interacts with *μ*‐opioid receptors, naloxone disrupts the transient shift in G‐protein binding by the μ‐opioid receptor from Gi/o to Gs. This disruption decreases opioid tolerance and dependence [41]. Furthermore, when administered at ultra‐low doses, naloxone escalates endorphin release by suppressing the negative feedback that occurs after the release of large quantities of enkephalins. This suppression ultimately increases antinociception and analgesia [42, 43]. Naloxone's dose‐dependent pain response has also been demonstrated in rats, with low doses inducing paradoxical analgesia and higher doses eliciting hyperalgesia [39, 44]. These findings suggest naloxone as a potential additive for anesthesia to enhance analgesia while reducing opioid use.

Our search revealed seven studies investigating ultra‐low‐dose naloxone as an additive in regional anesthesia. The included studies reported that the addition of naloxone at these doses to standard anesthetic drugs, with or without fentanyl, was associated with prolonged postoperative analgesia, increased duration of sensory and motor block, and decreased opioid consumption in both nerve block and IVRA conditions [6, 13, 14, 26, 27, 28, 29]. Additionally, patients administered naloxone during regional anesthesia were reported to have lower intraoperative and postoperative VAS scores [13, 14, 26, 29]. These patients also experienced better sleep quality than others; however, this was not significantly associated with pain severity [13, 26]. Notably, while Naloxone IV infusion did not enhance the analgesic qualities of IVRA or extend the duration of postanesthesia recovery, local naloxone administration could exert these effects [29]. Most studies have highlighted the potential of adjuvant naloxone in regional anesthesia.

In addition, included studies evaluated the impact of naloxone on postoperative complications [6, 26, 28]. The incidence of postoperative complications, such as PONV, pruritus, urinary retention, hypotension, bradypnea, injection pain, and chemosis, did not differ between patients who received naloxone and other groups [6, 13, 26]. The effect of higher doses of naloxone (in the range of micrograms and milligrams) on improving postoperative complications remains controversial [18, 20, 45, 46]. However, these findings should be interpreted cautiously because adverse events were generally uncommon, follow‐up periods were limited, and the total number of participants was relatively small. Therefore, the available evidence does not permit definitive conclusions regarding the overall safety profile of ultra‐low‐dose naloxone.

It is important to note that our study is based on limited clinical trials with small sample sizes. Moreover, there was variability in the dosing regimens of ultra‐low‐dose naloxone (50 vs. 100 ng), making it difficult to determine the optimal dose. None of the included studies investigated pediatric or adolescent populations, which limits the applicability of the findings to adults. Additionally, the timeframe for outcome assessment varied among the investigations, and the included studies demonstrated heterogeneity in surgical types, anesthetic regimens, and outcome measures.

Although the majority of studies were rated as having “good” methodological quality based on the NIH Quality Assessment Tool, several domains indicated potential sources of bias. Common methodological limitations included incomplete reporting of randomization procedures, lack of blinding, and insufficient information regarding outcome assessment. These gaps may have introduced performance or detection bias, affecting the validity of the findings. Additionally, the consistent reporting of positive results raises the possibility of publication bias, particularly given the limited number of included trials. Acknowledging these weaknesses is essential for accurately interpreting the pooled evidence and for highlighting the need for future well‐designed, transparently reported studies that adhere to rigorous methodological standards.

Besides, meta‐analysis was not applicable due to the heterogeneity of the included studies, the potential for publication bias, as well as the diversity of techniques, their anesthetic and analgesic mechanisms, and clinical implications. To achieve maximum efficacy without causing toxicity or complications, more clinical trials with larger sample sizes should be conducted to optimize the dose of naloxone administration in specific patient populations. Despite these limitations, our study provides valuable insights into the use of ultra‐low‐dose naloxone in regional anesthesia. This insight will help specialists in their clinical practice.

## 5 Conclusion

Naloxone is a *μ*‐receptor antagonist that can produce paradoxical effects at different doses. It exhibits opioid antagonist properties at higher doses and induces antinociceptive responses at nanogram dosage levels. The available evidence suggests that ultra‐low‐dose naloxone may be associated with prolonged postoperative analgesia and, in some studies, longer sensory and/or motor block duration and reduced postoperative opioid or analgesic consumption. However, these findings were not consistent across all studies or outcomes. The evidence base is limited to seven randomized controlled trials involving 564 adult patients, with substantial heterogeneity in anesthesia techniques, surgical procedures, naloxone dosing, and outcome assessment. Because quantitative meta‐analysis was not feasible, the observed findings should be interpreted as study‐level observations rather than definitive treatment effects. Although most included studies reported no significant differences in postoperative complications between naloxone and comparator groups, the available evidence is insufficient to establish the overall safety profile of ultra‐low‐dose naloxone. Larger, well‐designed randomized controlled trials with standardized outcome measures and adequate safety assessment are warranted to clarify its clinical efficacy and safety.

## Author Contributions

**Mohammadjavad Mehrabanian:** conceptualization, investigation, supervision. **Behrang Nooralishahi:** conceptualization, investigation, supervision, validation, methodology. **Mehdi Dehghani Firoozabadi:** conceptualization, investigation, supervision. **Mohammad Sahebjam:** conceptualization, investigation, supervision. **Yekta Ghane:** writing – original draft, writing – review and editing, data curation, project administration. **Homayoun Pishraft‐Sabet:** writing – original draft, writing – review and editing, data curation. **Nazila Heidari:** writing – original draft, writing – review and editing, project administration, data curation. **Akbar Shafiee:** conceptualization, investigation, writing – review and editing, methodology, supervision.

## Funding

The authors have nothing to report.

## Ethics Statement

The authors have nothing to report.

## Conflicts of Interest

The authors declare no conflicts of interest.

## Transparency Statement

Behrang Nooralishahi affirms that this manuscript is an honest, accurate, and transparent account of the study being reported; that no important aspects of the study have been omitted; and that any discrepancies from the study as planned (and, if relevant, registered) have been explained.

## Supporting information


**Table S1:** PRISMA 2020 checklist for reporting systematic reviews. **Table S2:** PRISMA 2020 checklist for abstracts of systematic reviews. **Table S3:** The list of search strategies and final results on each database. **Table S4:** The quality assessment of included clinical trials.

## Data Availability

Data sharing is not applicable to this article as no new data were created or analyzed in this study. All data in this study were obtained from publicly accessible sources. The datasets are included within the published article.
